# Impact of bias field correction on 0.35 T pelvic MR images: evaluation on generative adversarial network-based OARs’ auto-segmentation and visual grading assessment

**DOI:** 10.3389/fonc.2024.1294252

**Published:** 2024-03-28

**Authors:** Marica Vagni, Huong Elena Tran, Francesco Catucci, Giuditta Chiloiro, Andrea D’Aviero, Alessia Re, Angela Romano, Luca Boldrini, Maria Kawula, Elia Lombardo, Christopher Kurz, Guillaume Landry, Claus Belka, Luca Indovina, Maria Antonietta Gambacorta, Davide Cusumano, Lorenzo Placidi

**Affiliations:** ^1^ Dipartimento di Diagnostica per Immagini, Radioterapia Oncologica ed Ematologia, Fondazione Policlinico Universitario “A. Gemelli” IRCCS, Rome, Italy; ^2^ Mater Olbia Hospital, Olbia, Italy; ^3^ Department of Radiation Oncology, LMU University Hospital, LMU Munich, Munich, Germany; ^4^ German Cancer Consortium (DKTK), Partner Site Munich, A Partnership Between DKFZ and LMU University Hospital Munich, Munich, Germany; ^5^ Bavarian Cancer Research Center (BZKF), Munich, Germany

**Keywords:** N4ITK algorithm, bias field artifact, visual grading assessment, generative adversarial networks, 0.35 T MRIgRT, prostate cancer

## Abstract

**Purpose:**

Magnetic resonance imaging (MRI)-guided radiotherapy enables adaptive treatment plans based on daily anatomical changes and accurate organ visualization. However, the bias field artifact can compromise image quality, affecting diagnostic accuracy and quantitative analyses. This study aims to assess the impact of bias field correction on 0.35 T pelvis MRIs by evaluating clinical anatomy visualization and generative adversarial network (GAN) auto-segmentation performance.

**Materials and methods:**

3D simulation MRIs from 60 prostate cancer patients treated on MR-Linac (0.35 T) were collected and preprocessed with the N4ITK algorithm for bias field correction. A 3D GAN architecture was trained, validated, and tested on 40, 10, and 10 patients, respectively, to auto-segment the organs at risk (OARs) rectum and bladder. The GAN was trained and evaluated either with the original or the bias-corrected MRIs. The Dice similarity coefficient (DSC) and 95th percentile Hausdorff distance (HD95^th^) were computed for the segmented volumes of each patient. The Wilcoxon signed-rank test assessed the statistical difference of the metrics within OARs, both with and without bias field correction. Five radiation oncologists blindly scored 22 randomly chosen patients in terms of overall image quality and visibility of boundaries (prostate, rectum, bladder, seminal vesicles) of the original and bias-corrected MRIs. Bennett’s *S* score and Fleiss’ kappa were used to assess the pairwise interrater agreement and the interrater agreement among all the observers, respectively.

**Results:**

In the test set, the GAN trained and evaluated on original and bias-corrected MRIs showed DSC/HD95^th^ of 0.92/5.63 mm and 0.92/5.91 mm for the bladder and 0.84/10.61 mm and 0.83/9.71 mm for the rectum. No statistical differences in the distribution of the evaluation metrics were found neither for the bladder (DSC: *p* = 0.07; HD95^th^: *p* = 0.35) nor for the rectum (DSC: *p* = 0.32; HD95^th^: *p* = 0.63). From the clinical visual grading assessment, the bias-corrected MRI resulted mostly in either no change or an improvement of the image quality and visualization of the organs’ boundaries compared with the original MRI.

**Conclusion:**

The bias field correction did not improve the anatomy visualization from a clinical point of view and the OARs’ auto-segmentation outputs generated by the GAN.

## 1 Introduction

Magnetic resonance imaging-guided radiotherapy (MRIgRT) systems, specifically the combination of a linear accelerator (Linac) with an on-board MR scanner (MRI-Linac), provide the possibility to manage and effectively compensate anatomical changes that can occur between and within treatment sessions (1). This allows for the adaptation of the radiotherapy treatment plan on a daily basis, considering any changes in the patient’s anatomy. Additionally, on-board MRI, offering high soft tissue contrast (2), enables good visualization of anatomical structures, facilitating accurate delineation of organs at risk (OARs) and target volumes (3, 4), without additional dose for imaging purposes.

However, smooth, low-frequency variations in signal intensity across the image known as bias field artifact can occur, resulting in signal losses that may affect MR image quality (5, 6).

This artifact arises from various sources, including sensitivity variations of the imaging system, magnetic field inhomogeneities, and patient-related factors (6). It manifests as a gradual change in signal intensity, resulting in a non-uniform intensity distribution across the image that can obscure important structures, reduce contrast, and compromise the accuracy of image analysis techniques (5, 6). The presence of the bias field artifact might pose challenges in tasks demanding accurate and quantitative measurements, such as radiomics, segmentation, and registration. Additionally, the artifact could impact the effective deployment of artificial intelligence systems, including those utilizing deep learning neural networks. It can introduce errors and inaccuracies in the measurements, potentially impacting diagnostic and treatment decisions (5).

Several methods have been developed to address bias field artifacts (5); the most effective and used technique relies on the N4 bias field correction (N4ITK) algorithm (7, 8), an extension of the N3 algorithm (6) specifically designed to tackle bias field correction in MRI. By employing a multiresolution non-parametric approach, the N4ITK algorithm deconvolves the histogram of the intensities of the original corrupted image by using a Gaussian function, estimates the “corrected” intensities, and spatially smooths the resulted bias field estimation using a B-spline model (7).

High-quality images are also crucial for accurate image interpretation, as fine details, subtle abnormalities, and specific characteristics of the disease can be better observed, reducing the risk of misdiagnosis and leading to more precise treatment plans (9).

The aim of this study was to assess the impact of the bias field correction applied to 0.35 T pelvis MRIs in both anatomy visualization from a clinical point of view and quantitative application such as the OARs’ auto-segmentation output generated by a generative adversarial network (GAN). To the best of our knowledge, no studies have systematically investigated the impact of the bias field artifact at 0.35 T, as well as in neural network auto-segmentation tasks.

## 2 Materials and methods

### 2.1 Dataset

The cohort of patients retrospectively enrolled was composed of 60 prostate cancer subjects who underwent MRIgRT (MRIdian, ViewRay, Mountain View, USA) from August 2017 to September 2022 at Fondazione Policlinico Universitario “A. Gemelli” IRCCS (FPG) in Rome, Italy.

MR images were acquired with a True Fast Imaging with Steady-state Precession (TrueFISP) sequence on a 0.35-T MRI scanner in free breathing condition, resulting in T2*/T1 contrast images with a spacing of 1.5 × 1.5 × 1.5 mm.

The delineations of the OARs rectum and bladder were provided and represented our ground truth to train and evaluate a neural network able to auto-segment them (Section 2.4). To ensure consistency, a radiation oncologist with over 5 years of experience in 0.35 T MRI pelvic examination reviewed and adjusted the delineations.

The dataset was randomly split into training (40 patients), validation (10 patients), and testing (10 patients) sets for the neural network application (Section 2.4), while a subset of 22 randomly selected patients was considered in order to assess the impact of the bias field correction on the visual inspection of the patients’ anatomy using a visual grading assessment (VGA, Section 2.3).

### 2.2 Bias field correction: N4ITK algorithm

As the first preprocessing step, the N4 bias field correction algorithm (N4ITK) (7) was applied to remove inhomogeneity artifacts affecting the MRIs. To fit the algorithm on our 0.35 T MRIs, optimization of the input parameters was performed by varying the number of fitting levels (number of hierarchical resolution levels to fit the bias field) into [3, 4, 5] and the number of control points (number of points defining the B-spline grid for the first resolution level) into [4, 6, 8]. The chosen parameter set was configured with three fitting levels and six control points (more details in the **Supplementary Materials**, Section 1). The algorithm requires an image mask to be supplied by the user to indicate the voxels considered to estimate the bias field: the one identifying the patient’s body was used. The number of iterations was set equal to 100. For the other required parameters, the default values were kept (7). The N4ITK algorithm implemented in Python within the SimpleITK toolkit was used for this study.

The performance of the algorithm with the chosen parameters was also visually assessed on an external dataset of 0.35 T pelvic MRIs provided by the Department of Radiation Oncology of the University Hospital of the LMU in Munich, Germany (**Supplementary Materials**, Section 1).

### 2.3 Evaluation of artifact impact: visual grading assessment

Effective methods of assessing image quality and the output of an image processing technique in the clinical scenario rely on the VGA, where observers/raters visually grade a certain characteristic of the image (10). In order to assess the impact of the bias field correction on the visual inspection of the patients’ anatomy, an absolute VGA on a subset of 22 randomly chosen patients was performed. Five radiation oncologists with varying levels of experience in 0.35 T MRI pelvic examination were identified. Observers O1 and O2 work at Fondazione Policlinico Universitario “A. Gemelli” IRCCS, Rome; observers O3, O4, and O5 work at Mater Olbia Hospital, Olbia. Observer O5 has the most experience in conducting prostate cancer examinations.

The raters blindly scored the selected patients in terms of overall image quality and visibility of the boundaries of clinically relevant structures (i.e., prostate, bladder, rectum, and seminal vesicles), using a 4-point and a 5-point visual grading analysis scale, respectively (details in **Table 1**). The grading was carried out on both the original and the bias-corrected MRI volumes. For the assessment of the seminal vesicles, 20 out of 22 patients were considered since the structures were not visible due to the clinical history of the subjects.

**Table 1:** Overview of grading scales used for the assessment of overall image quality and visibility of the structures. The first column represents the score.

OVERALL IMAGE QUALITY *Criterion: image quality in terms of visualization of the area of interest for the clinical purpose (contouring for radiotherapy treatment)*			
1	POOR	Significant presence of noise/artifacts, which compromise the interpretation of the image in the area of interest.	
2	ACCEPTABLE	Acceptable quality, presence of noise/artifacts which however do not compromise the visualization of the area of interest. Suitable for the clinical purpose (contouring for radiotherapy treatment). Improvements are desired.	
3	GOOD	Good quality, absence of noise/artifacts that can compromise the use of the image for the clinical purpose (contouring for radiotherapy treatment).	
4	EXCELLENT	Excellent quality. No noise/artifacts.	
STRUCTURE VISIBILITY *Criterion: clear visualization of the structure of interest and its boundaries, for contouring*			
1	Criterion absolutely not fulfilled (not visible)		Structure’s boundaries not visible.
2	Criterion probably not fulfilled (unclear)		Structure’s boundaries partially visible, but not completely.
3	Undecided		Observer undecided on the score to assign.
4	Criterion quite fulfilled (clear)		Structure’s boundaries visible, but their definition can be improved.
5	Criterion fulfilled (very clear)		Structure’s boundaries clearly visible.

The five observers participated independently in the visual assessment. The MRI volumes were presented randomly to the graders, and they were allowed to freely adjust the window level of the image intensities in order to reproduce the clinical scenario.

After collecting the absolute ratings, they were converted into relative ones by subtracting the score given to the bias-corrected MRI from the score given to the original MRI. In this way, we could compare whether the observers perceived or not an improvement after the artifact correction, independently from the absolute grade associated with the MR volume.

Subsequently, the *S* score proposed by Bennett et al. (11) was used to assess the pairwise interobserver agreement, while Fleiss’ kappa (12) was used to assess the interrater agreement among all the observers. Both metrics come from the general kappa agreement score (13), given by:


$$k=\frac{P_{\text{o}}-P_{\text{e}}}{1-P_{\text{e}}}$$

where $P_{\text{o}}$ is the observed proportion of agreement and $P_{\text{e}}$ is the proportion of agreement that could be expected on the basis of chance. Therefore, the scores’ values range from −1 to 1, where 1 indicates perfect agreement, 0 is exactly what would be expected by chance, and negative values indicate agreement less than the chance (potential systematic disagreement between the observers) (14). Statistical analyses were performed using Python statistical analysis packages and R (version 4.2.3).

### 2.4 Evaluation of artifact impact: generative adversarial network

The neural network implemented in this study was adapted from the Vox2Vox Generative Adversarial neural network, first proposed by Cirillo et al. (15). While various network architectures for medical imaging segmentation have shown promising results, one of the main advantages of using a GAN architecture is that the discriminator network also acts as a shape regulator by discarding output segmentations that do not look realistic (16, 17). In many cases, GANs produce more refined segmentation results, which conventional U-Nets can achieve by post-processing techniques, with an increased computational complexity (17). For example, Wang et al. (18) reported superior performance of the GAN architecture over conventional U-Net for automatic prostate segmentation on MRI.

The proposed 3D GAN was trained separately for each OAR (rectum, bladder), once by giving as input the original MRI volumes and once by giving the bias-corrected MRI volumes. Therefore, we obtained four networks: two trained and evaluated using the original images and two using the bias-corrected ones, for the bladder and the rectum, respectively. Hyperparameters’ tuning was carried out and the best set of parameters for each network was set based on the performance evaluated over the validation set. Auto-segmentation of each OAR was then performed, once given as input the original and once the bias-corrected MRI volumes. Specifications about the GAN implementation can be found in the **Supplementary Materials**, Section 2.

The quantitative evaluation of the OARs’ auto-segmentation was carried out by computing the Dice similarity coefficient (DSC) and the 95th percentile Hausdorff distance (HD95^th^) (19) between the generated and the ground-truth delineations for the testing set, for both the configuration with the original and the bias-corrected MRI volumes. The statistical significance of the difference between the metrics computed for the two groups was assessed by using the Wilcoxon signed-rank test (significance level set at 0.05).

## 3 Results

### 3.1 N4ITK application

**Figure 1:**
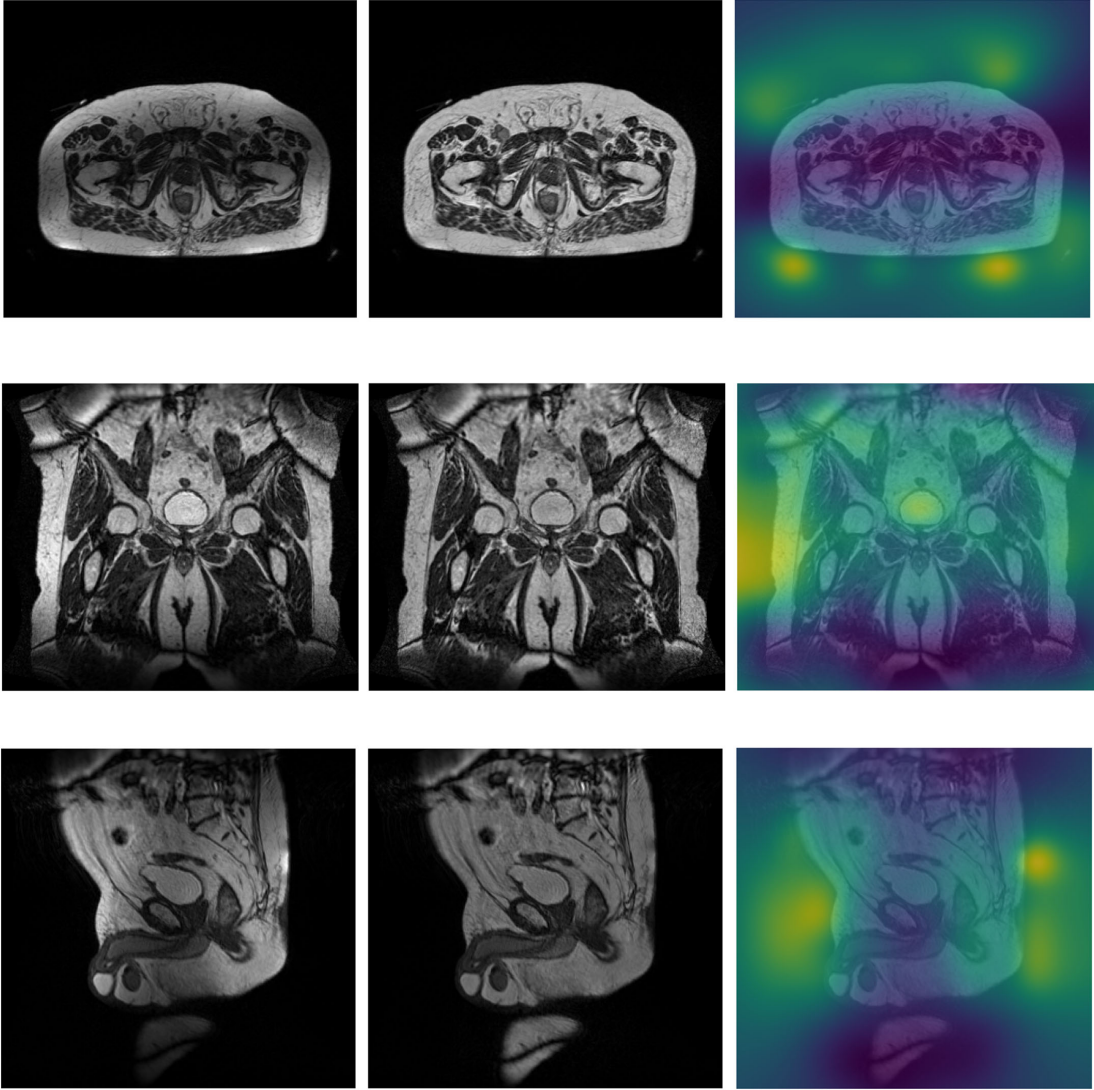
Axial (first row), coronal (central row), and sagittal (bottom row) central slice from the original MRI volume (left column), the bias-corrected MRI volume (central column), and the estimated bias field map with the chosen combination of N4ITK parameters (right columns), for a randomly selected patient. The darker region in bias field estimation (right column) indicates where the bias field is higher.

**Figure 1** shows the central slice from the original MRI volume and the corrected one using the N4ITK algorithm in the axial, coronal, and sagittal views, along with the estimated bias fields, for a randomly selected patient.

### 3.2 Evaluation of artifact impact: visual grading assessment

**Figure 2:**
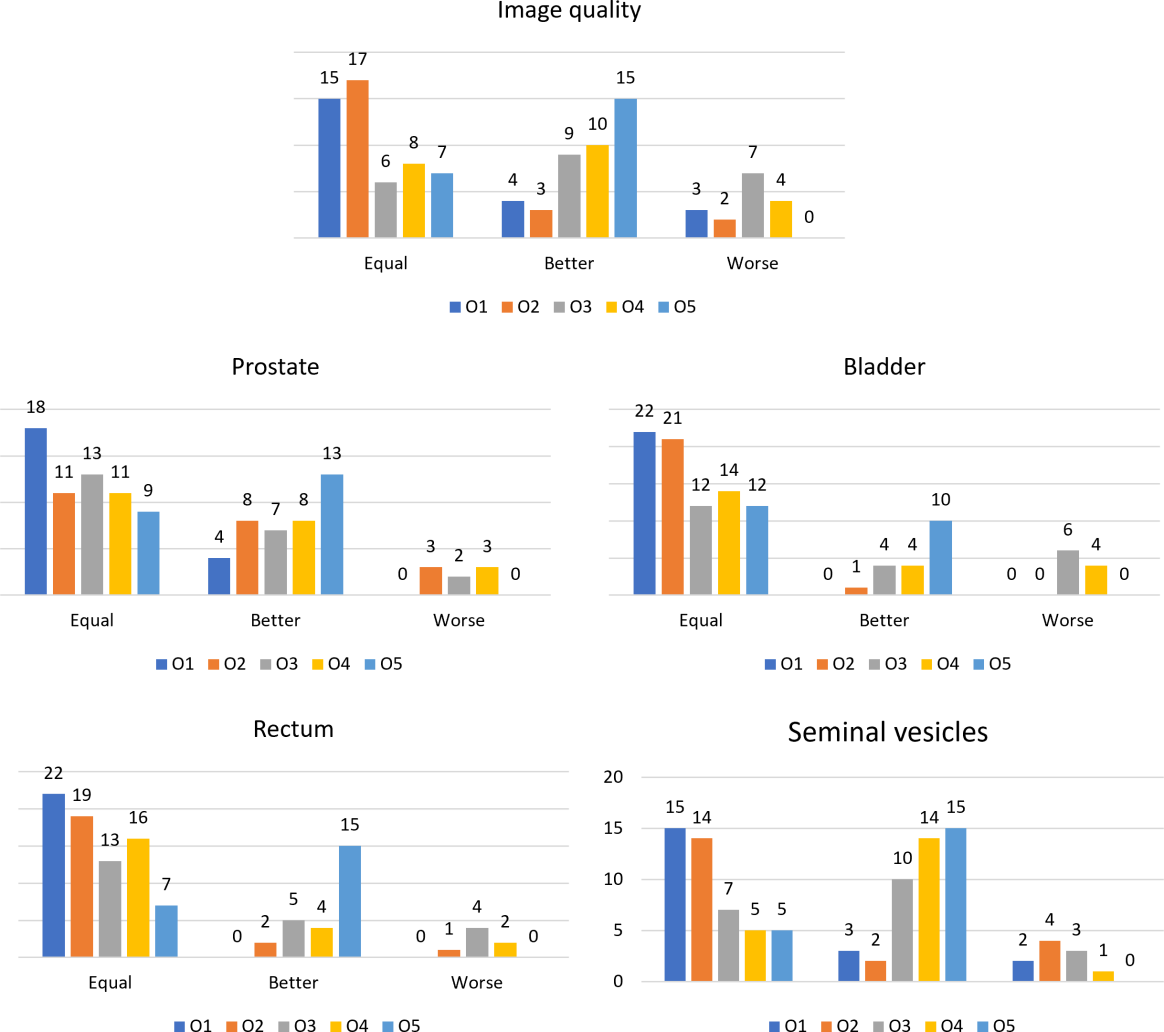
Bar plots representing the number of patients scored as equal, better, or worse in the comparison between the original MR volume and the bias-corrected one, for the different observers for the considered anatomical structures and the overall image quality.

The scores resulting from the VGA are presented in **Figure 2**. Bar plots show the number of patients scored as being equal, better, or worse in terms of image quality and visibility of the boundaries of anatomical structures after applying the N4ITK algorithm to the original MR volumes.

**Figure 3:**
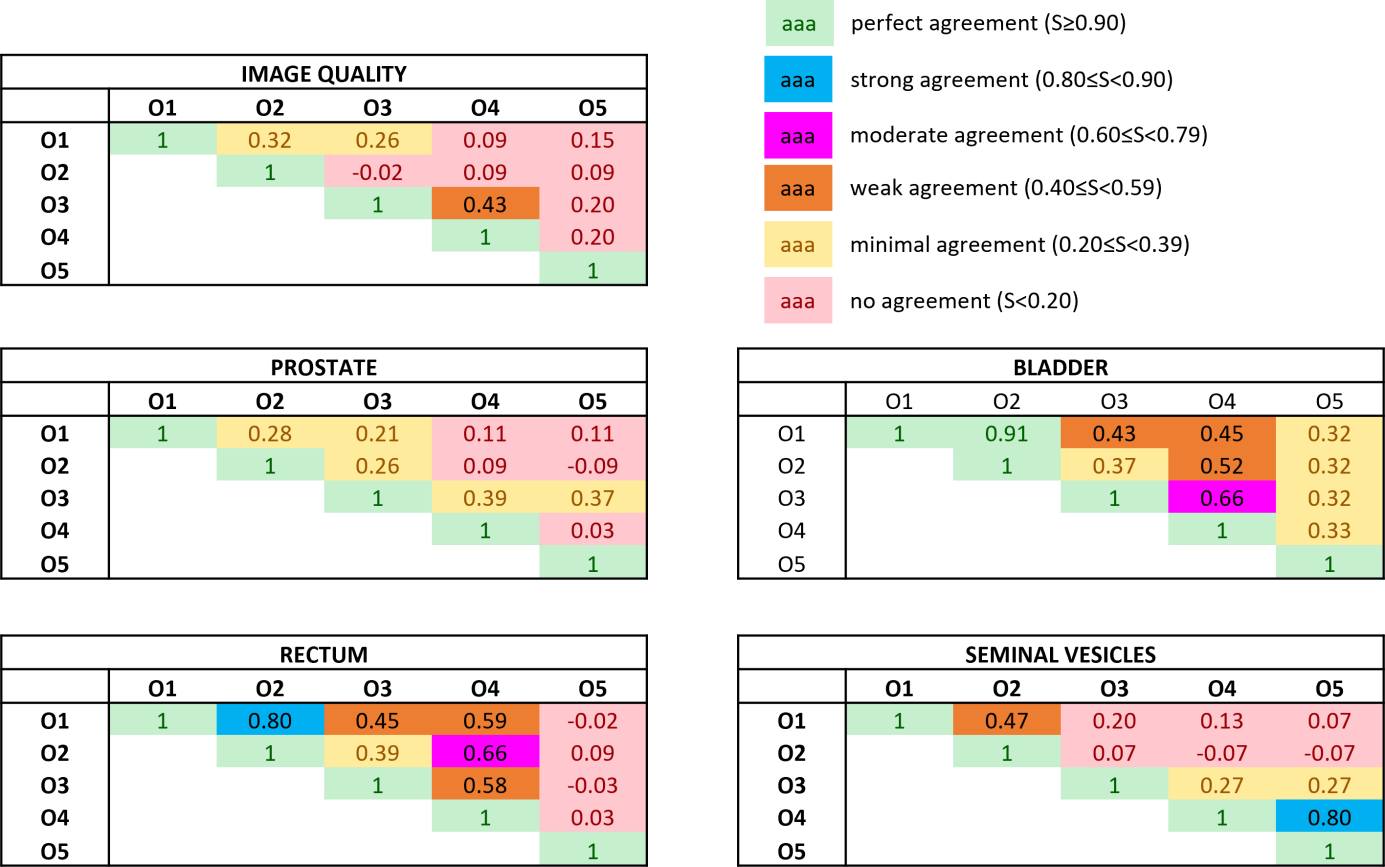
Bennett’s *S* score assessing the pairwise interobserver agreement in rating both the overall image quality and the anatomical structures’ visibility. The ratings were obtained by subtracting the absolute score between the bias-corrected MR volume and the original MR volume. Colors give a snapshot of the agreement: (green) perfect agreement (*S* ≥ 0.90), (blue) strong agreement (0.80 ≤ *S*< 0.90), (magenta) moderate agreement (0.60 ≤ *S*< 0.79), (orange) weak agreement (0.40 ≤ *S*< 0.59), (yellow) minimal agreement (0.20 ≤ *S*< 0.39), (red) no agreement (*S*< 0.20).

The Bennet’s score analyzing the agreement for pairwise observers is reported in **Figure 3**. The relative Fleiss’ kappa assessing the interrater agreement among all the observers for the image quality was equal to −0.02, while for the visibility of the boundaries of prostate, bladder, rectum, and seminal vesicles, the metric scored −0.07, 0.08, −0.02, and 0.06, respectively.

### 3.3 Evaluation of artifact impact: GAN’s performance

**Figure 4:**
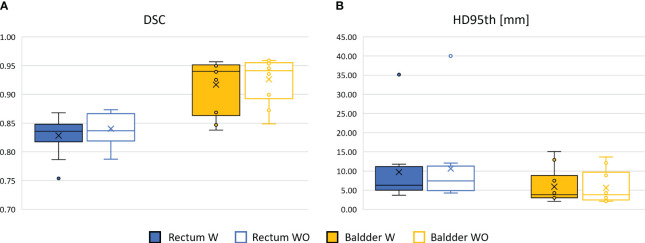
Comparison between the performance of the 3D GAN in delineating the OARs in terms of DSC box **(A)** and HD95th box **(B)** distributions for the patients included in the test set, when it is trained and evaluated on a dataset with (W, color-filled boxes) or without (WO, empty boxes) bias field correction. Boxplots are included in quartile values; the horizontal line indicates the median value, and the “x” is the mean value. The HD95th is given in millimeters.

**Figure 5:**
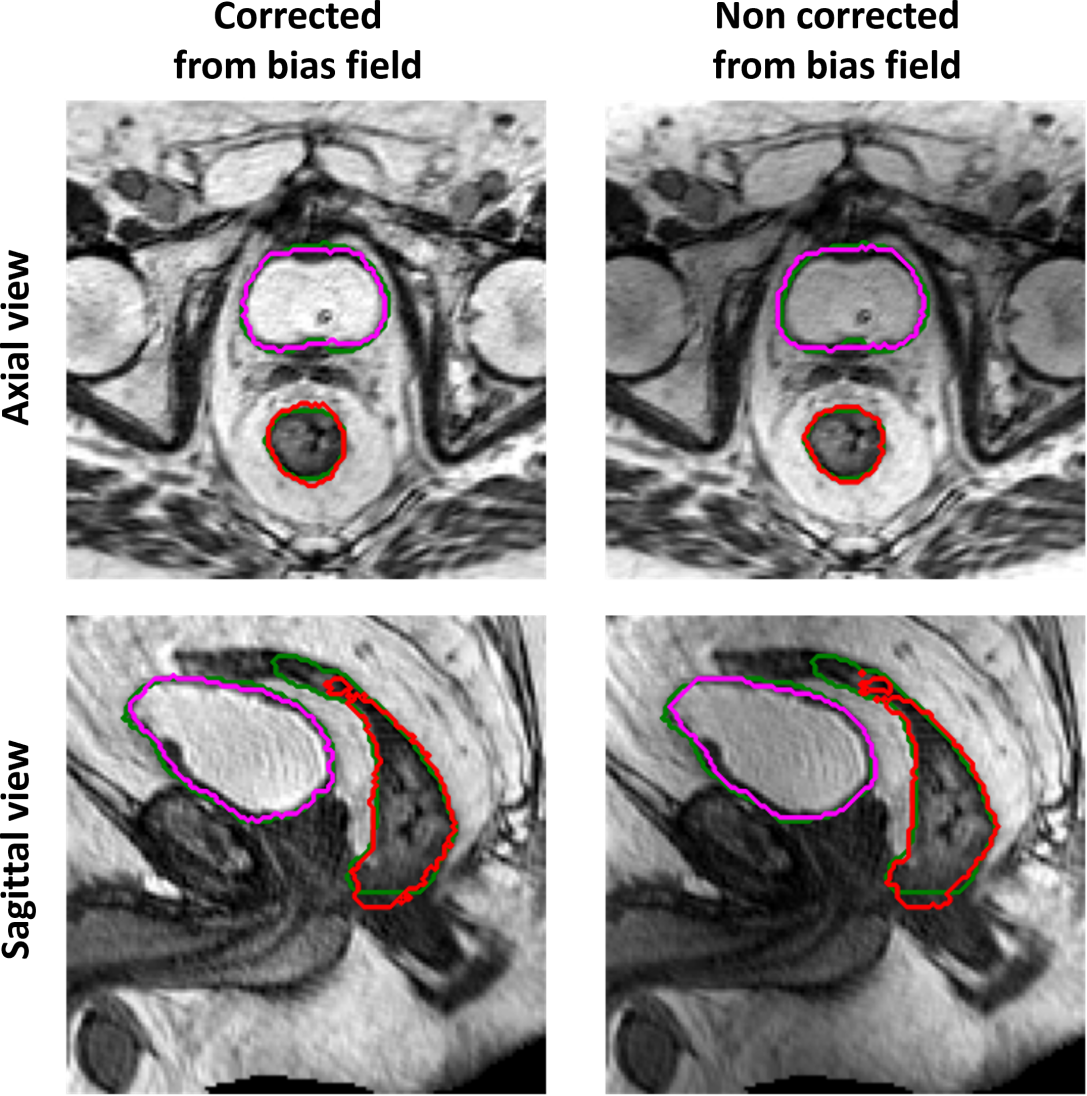
Example of ground-truth (green) and generated segmentations of the rectum (red) and bladder (magenta), in the axial (top row) and sagittal (bottom row) view, for a test set patient. The left column illustrates the results obtained when the GAN is trained and evaluated on a dataset corrected from bias field; the right column illustrates the results obtained when the GAN is trained and evaluated on the original dataset (without bias field correction).

The performance of the 3D GAN for the contours automatically generated when feeding the neural network with the original or the bias-corrected MRI was compared in the test set using the evaluation metrics. **Table 2** summarizes the results in terms of metrics averaged across the patients in the test set, while **Figure 4** illustrates the metrics’ distribution across the test set: no statistical differences were found neither for the bladder (*p* = 0.07 and *p* = 0.35 for the DSC and the HD95^th^, respectively) nor for the rectum (*p* = 0.32 and *p* = 0.63 for the DSC and the HD95^th^, respectively). An example of the OARs’ contours generated by the neural network trained and evaluated by using both original and bias-corrected MRIs is illustrated in **Figure 5**.

**Table 2:** Comparison between the performance of the 3D GAN in delineating the OARs, averaged across all the patients in both the case with and without the bias field correction. Standard deviations from the mean values (std) are also reported. The column “Time” refers to the average time required by the network to generate a new segmentation volume for each patient.

Organ	With bias field correction			Without bias field correction		
	DSC (± std)	HD95^th^ (± std) [mm]	Time (± std) [s]	DSC (± std)	HD95^th^ (± std) [mm]	Time (± std) [s]
*Bladder*	0.92 (0.05)	5.91 (4.53)	1.29 (0.13)	0.92 (0.05)	5.63 (4.30)	1.26 (0.14)
*Rectum*	0.83 (0.03)	9.71 (9.31)	1.15 (0.11)	0.84 (0.03)	10.61 (10.70)	1.17 (0.22)

## 4 Discussion

This study explored whether bias field correction performed over 0.35 T pelvic MRIs improves the automatic segmentation of pelvic OARs and enhances anatomy visualization during clinical practice. Regarding the VGA, the application of the N4ITK algorithm with respect to the original MR volume resulted mostly in either no change or an improvement of the image quality perception and visualization of the boundaries of relevant clinical structures. Concerning the performance of GAN, the bias field correction did not improve the computed evaluation metrics.

The results obtained in terms of evaluation metrics averaged over the patients in the test set (**Table 2**; **Figure 4**) show that the GAN model is not improving despite the bias field correction. The reasons why there is no improvement could be several: firstly, the patients in the development set are heterogeneous in terms of bias field presence, so the network may learn to isolate this characteristic [similar to performing data augmentation (20, 21)]), showing its robustness in compensating the presence of an artifact in its input data. In addition, the artifact uniformly affects the region of interest of the dataset in this study; thus, the segmentation task might not be affected by the effect of the bias field since the contrast is not much altered. Adequate contrast between different regions and structures in an image is important to distinguish objects and boundaries, making auto-segmentation tasks easier. Other applications, such as reconstruction or image enhancement, could be further improved by preprocessing steps like bias field correction.

From the VGA analysis, overall, no changes or improvements of the evaluated criteria before and after the bias field correction were observed (**Figure 2**). One of the reasons for no improvement in the visualization could be that, while visually assessing the MR volumes, the graders had the possibility to change the window width and window level of the image intensities. In this way, the observers could “correct” the image visualization and perhaps mitigate the artifact effect.

The values of the Fleiss’ kappa close to zero show that there was no agreement among all the readers in grading the MRIs’ image quality and anatomical structures’ visibility patient-wise. However, from **Figure 2**, a certain agreement between observers O1 and O2 and O3 and O4 can be appreciated, particularly for the bladder and rectum evaluation. This is also true for Bennett’s score results reported in **Figure 3**: observers O1 and O2 reached perfect (*S* = 0.91) and strong (*S* = 0.80) agreement in the bladder and rectum evaluation, respectively. On the other hand, observers O3 and O4 reached moderate agreement (*S* = 0.66) for the bladder evaluation, but weak agreement (*S* = 0.58) for the rectum. Observer O5 is mainly in disagreement with the others, except for the seminal vesicles where he reached a strong agreement with O4 (*S* = 0.80). Observer O5 is the one who appreciated more the correction of the artifact.

Considering the overall image quality, Bennett’s *S* score ranged from no agreement to weak agreement, showing that the pairwise agreement between the observers was slightly higher than what would be expected by chance. This result could arise from the fact that the MRI quality can also be affected by other artifacts not considered in this study, interfering with the evaluation of the observers. A similar situation can be appreciated for the evaluation of the prostate’s boundaries: the scores show no or minimal agreement in assessing the organ, indicating weak consistency between raters’ observations. The prostate is indeed considered one of the most challenging organs to assess during the visualization of prostate cancer patients (22).

Generally speaking, it may happen that the agreement between different observers is not very high in the patient-wise evaluation of the criteria: it is well known that the visualization, and therefore the delineation, of the structures of interest is affected by inter- and intraoperator variability (23–26).

This study has a limitation due to the absence of an assessment of intraobserver variability. As a result, we cannot guarantee consistent scoring by the same observer for identical images. Consequently, the outcomes may have been influenced by this potential variability.

In conclusion, GAN was robust to the variations in the signal caused by the bias field artifact, and therefore, it was able to isolate the effect and to auto-segment the OARs with the same accuracy for both the corrected and uncorrected MR volumes. In addition, the bias field’s presence did not compromise the anatomical interpretation of the clinicians; however, this outcome might be attributed to the challenge of visually detecting the gradual shading across the image caused by the artifact. We believe that while the impact of bias field correction in training a neural network for auto-contouring or in improving the image quality from a clinical perspective was not substantial, it could still serve as a useful tool for radiation oncologists in challenging contouring cases (**Supplementary Figure 3**), thus representing a valuable option for future MRI-Linac releases. Further studies will assess the impact of other preprocessing techniques in auto-segmentation, as well as in other tasks such as reconstruction or image enhancement. Moreover, we will assess the impact of the image artifacts during the course of the treatment.

## Data availability statement

The original contributions presented in the study are included in the article/**Supplementary Material**. Further inquiries can be directed to the corresponding author.

## Ethics statement

The studies involving humans were approved by the ethics committee (authorization number 3460). The studies were conducted in accordance with the local legislation and institutional requirements. Written informed consent for participation was not required from the participants or the participants’ legal guardians/next of kin in accordance with the national legislation and institutional requirements.

## Author contributions

MV: Writing – original draft, Conceptualization, Formal analysis, Methodology, Software. HT: Writing – review & editing, Methodology. FC: Writing – review & editing, Investigation. GC: Writing – review & editing, Investigation. AD’A: Writing – review & editing, Investigation. ARe: Writing – review & editing, Investigation. ARo: Writing – review & editing, Investigation. LB: Writing – review & editing, Supervision. MK: Writing – review & editing. EL: Writing – review & editing. CK: Writing – review & editing. GL: Writing – review & editing. CB: Writing – review & editing, Supervision. LI: Writing – review & editing, Supervision. MG: Writing – review & editing, Supervision. DC: Writing – review & editing, Methodology, Supervision. LP: Writing – review & editing, Methodology, Supervision.
