# Supplementary material for: Impact of bias field correction on 0.35 T pelvic MR images: evaluation on generative adversarial network-based OARs’ auto-segmentation and visual grading assessment
Source: Front Oncol. 2024 Mar 28;14:1294252. doi: 10.3389/fonc.2024.1294252 (PMC11007142; doi:10.3389/fonc.2024.1294252)
Supplement: Supplementary file 1 [file DataSheet_1.pdf]

Supplementary Material

Impact of bias field correction on 0.35 T pelvic MR images: evaluation on Generative Adversarial Network-based OARs auto-segmentation and Visual Grading Assessment

Marica Vagni, Huong Elena Tran, Francesco Catucci*, Giuditta Chiloiro, Andrea D'Aviero, Alessia Re, Angela Romano, Luca Boldrini, Maria Kawula, Elia Lombardo, Christopher Kurz, Guillaume Landry, Claus Belka, Luca Indovina, Maria Antonietta Gambacorta, Davide Cusumano†, Lorenzo Placidi†

†These authors share last authorship

*** Correspondence:** Dr. Francesco Catucci: francesco.catucci@materolbia.com

1 N4ITK specifications

After tuning the parameters defining the characteristics of the N4ITK algorithm, the estimated bias field maps before and after the correction were inspected (example illustrated in Supplementary Figure 1).

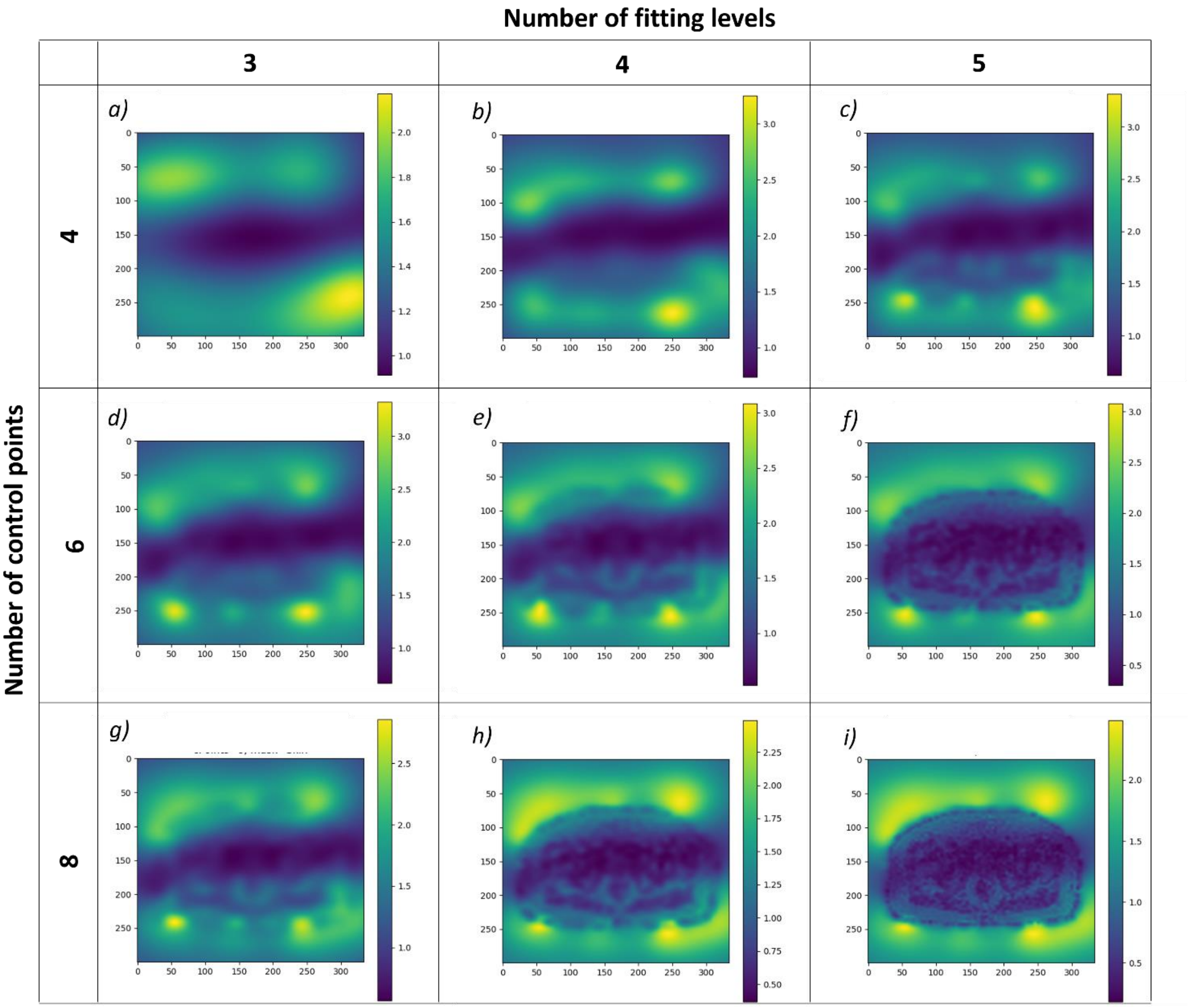

Supplementary Figure 1: Bias field map after N4ITK algorithm application for different configurations of the input parameters. Columns from left to right: number of fitting levels equal to 3, 4 and 5. Rows from top to bottom: number of control points equal to 4, 6 and 8. The plots refer to the axial central slice of an exemplary patient from the internal dataset (Fondazione Policlinico Universitario "A. Gemelli" IRCCS in Rome, Italy). Darker regions in the bias field estimation indicate where the bias field is higher.

The chosen set was therefore configured with 3 fitting levels and 6 control points (Supplementary Figure 1d). As it can be seen in Supplementary Figure 1, in configurations (c), (e), (f), (g), (h) and (i)

the N4ITK algorithm is overfitting the inhomogeneities of the internal anatomy rather than the smooth and low-frequency inhomogeneities caused by the bias field artifact: for this reason, they were not taken into consideration. Between the remaining Supplementary Figure 1 parameters' set (a), (b) and (d), configuration (d) was chosen as the best one based on visual inspection of the images obtained after bias field correction.

The same procedure was applied to an external set of 0.35T pelvic MRIs provided by the Department of Radiation Oncology of the University Hospital of the LMU in Munich, Germany. We visually assessed that the performance is equivalent to that in the internal dataset. Supplementary Figure 2 illustrates the results obtained from a representative patient.

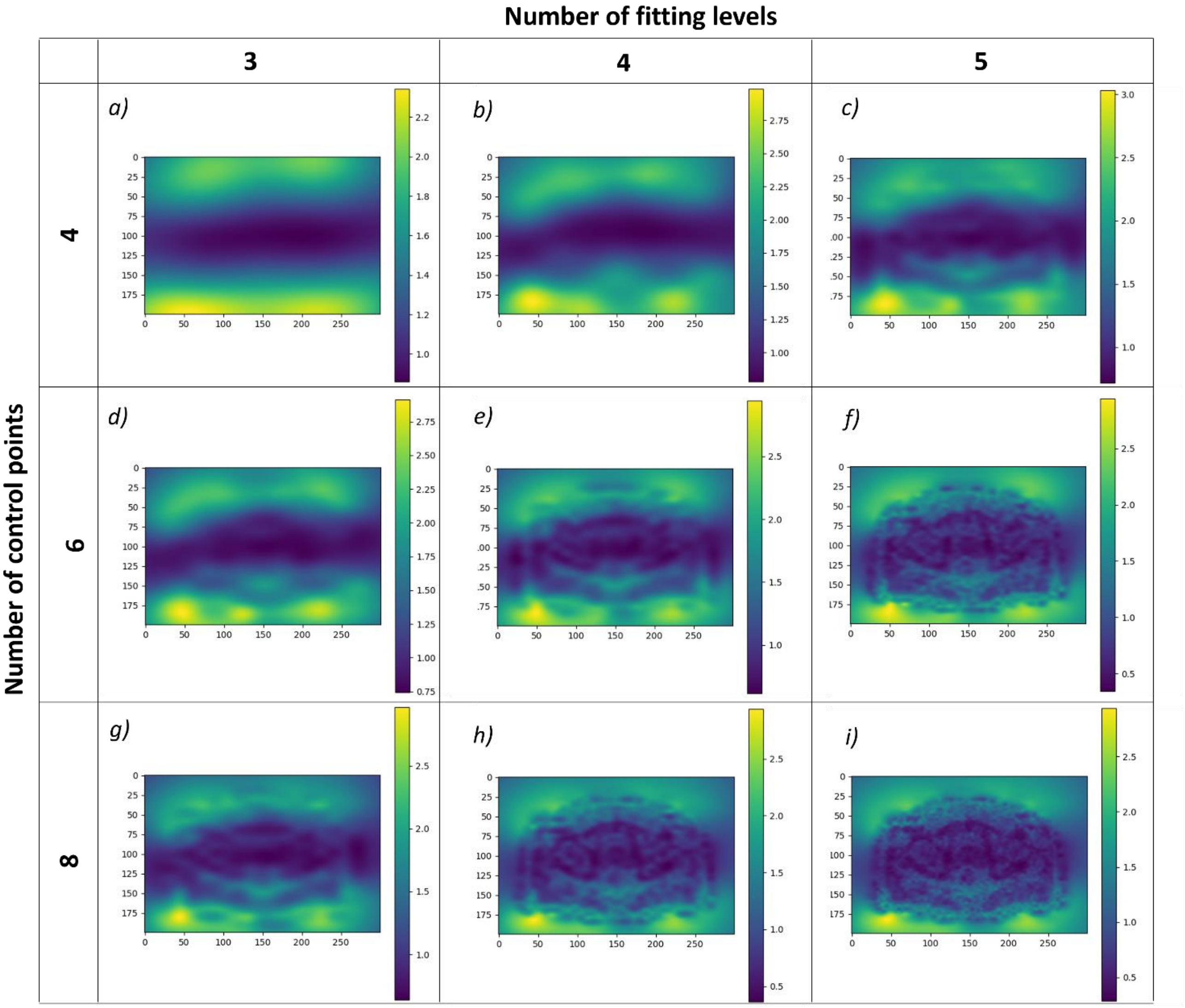

Supplementary Figure 2: Bias field map after N4ITK algorithm application for different configurations of the input parameters. Columns from left to right: number of fitting levels equal to 3, 4 and 5. Rows from top to bottom: number of control points equal to 4, 6 and 8. The plots refer to the axial central slice of an exemplary patient from the external dataset (Department of Radiation Oncology of the University Hospital of the LMU in Munich, Germany). Darker regions in the bias field estimation indicate where the bias field is higher.

Supplementary Figure 3 depicts the axial central slice of the original MRI volume and the bias-corrected one using the N4ITK algorithm for a challenging patient in order to showcase the effectiveness of bias-field correction.

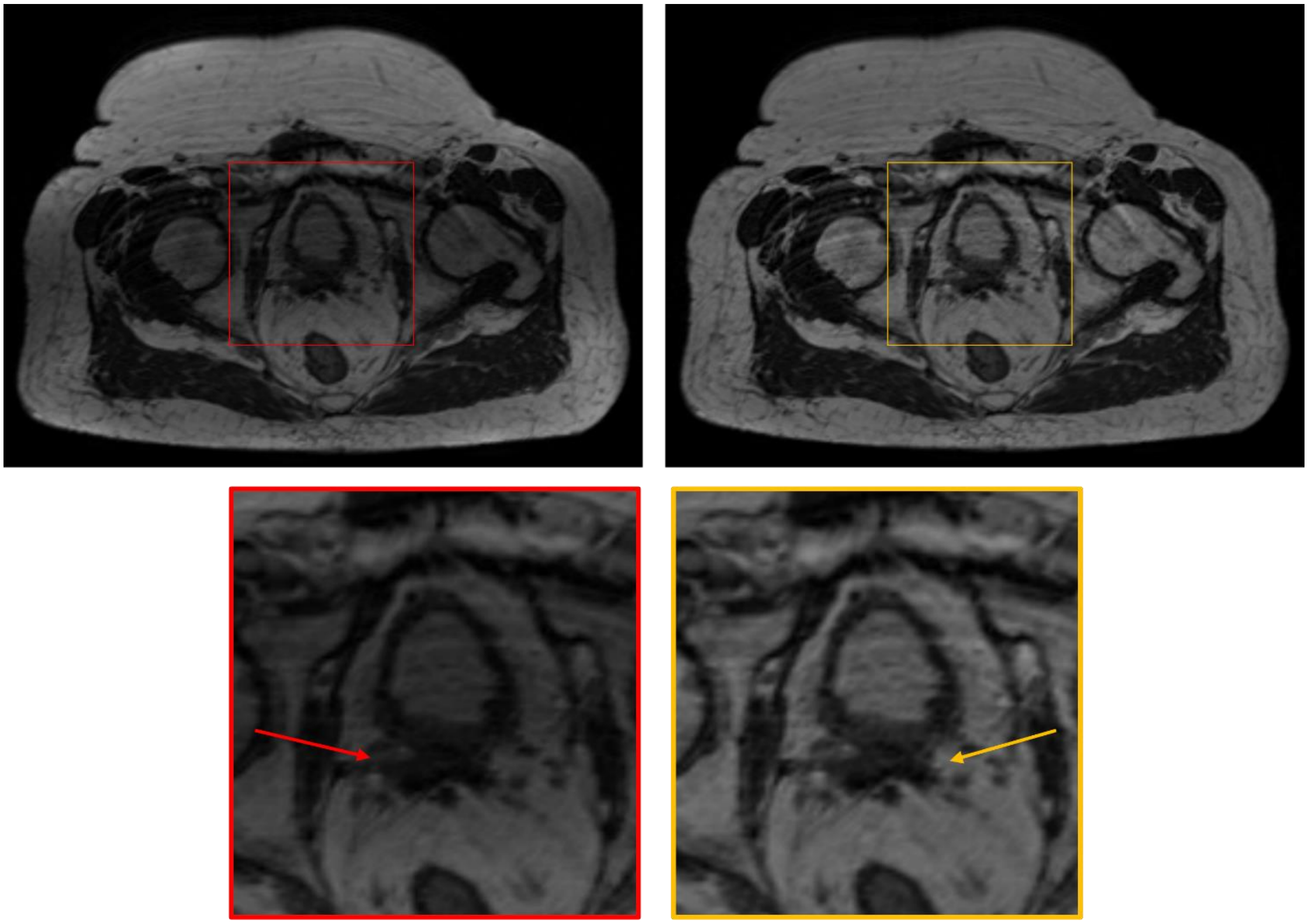

Supplementary Figure 3: Axial central slice from the original MRI volume (left column) and the bias-corrected MRI volume (right column) representing a challenging case demonstrating the effectiveness of bias-field correction. In fact, the seminal vesicles are obscured in the original slice (red arrow) but become visible after the bias-field correction (yellow arrow).

2 Generative Adversarial Network (GAN) specifications

The neural network implemented in this study consists of two sub neural networks: the generator, trained to generate from an input MR volume the correspondent OAR segmentation volume, and the discriminator, trained to distinguish the real from the generated OAR binary masks. The training was driven by the minimization of the generator loss, whose goal is to produce segmentations as similar (by minimizing the L2 distance) and overlapped (by maximizing the Dice Similarity Coefficient) as possible to the ground-truth ones. The batch size was set to 1. The network's weights have been updated iteratively by means of the Adaptive Moment Estimation (ADAM) algorithm with $\boldsymbol{\beta_1}$ and $\boldsymbol{\beta_2}$ parameters set to 0.5 and 0.999, respectively. Hyperparameters' tuning was carried out and the best set of parameters for each network was set as summarized in Supplementary Table 1, for each OARs.

Supplementary Table 1: Chosen hyperparameters for the different network configurations trained for each organ of interest.

	Generator learning rate	*Discriminator learning rate*	*Epoch decay start*	*Decay rate*	λ*
Bladder	0.001	0.0001	None	None	10
Rectum	0.001	0.0001	400	0.9	100

**scalar weight coefficient multiplying the Generator's loss.*

To prepare data for the neural network training and evaluation, each MRI volume and the correspondent OARs' binary masks were cropped to 128x128x128 pixels, while the grid size of 1.5 mm^3 was kept. MRI intensities were standardized by subtracting the mean value and dividing by the standard deviation of the patient volume, and then normalized to the range of [0,1] using min-max scaling between the 1st and 99th percentile of the patient volume.

The predicted mask obtained as network's output was binarized by applying a threshold equal to 0.5. To refine the output segmentation, in case of presence of multiple segmented regions in a single slice,

only the region with the largest area was kept, while segmented regions consisting of a single pixel were considered noise and thus deleted.

Neural networks were implemented with PyTorch (Python 3.8). GAN training and optimization was performed on an NVIDIA-Quadro-RTX-5000-GPU with 16GB of RAM, while the GAN evaluation was executed on an Intel(R)-Xeon(R)-Gold-6238T CPU@1.90GHz.
